# Geographic distribution of amino acid mutations in DHFR and DHPS in *Plasmodium vivax* isolates from Lao PDR, India and Colombia

**DOI:** 10.1186/s12936-016-1543-8

**Published:** 2016-09-21

**Authors:** Naowarat Saralamba, Supatchara Nakeesathit, Mayfong Mayxay, Paul N. Newton, Lyda Osorio, Jung-Ryong Kim, Nicholas J. White, Nicholas P. J. Day, Arjen M. Dondorp, Mallika Imwong

**Affiliations:** 1 Department of Molecular Tropical Medicine and Genetics, Faculty of Tropical Medicine, Mahidol University, Bangkok, Thailand; 2 Mahidol-Oxford Tropical Medicine Research Unit, Faculty of Tropical Medicine, Mahidol University, Bangkok, Thailand; 3 Lao-Oxford-Mahosot Hospital-Wellcome Trust Research Unit (LOMWRU), Microbiology Laboratory, Mahosot Hospital, Vientiane, Lao People’s Democratic Republic; 4 Faculty of Postgraduate Studies, University of Health Sciences, Vientiane, Lao People’s Democratic Republic; 5 Centre for Tropical Medicine and Global Health, Churchill Hospital, University of Oxford, Oxford, UK; 6 International Centre for Medical Research and Training (CIDEIM), Cali, Colombia; 7 Centre for Tropical Medicine & Parasitology, Kolkata, India

**Keywords:** *Plasmodium vivax*, Sulfadoxine–pyrimethamine, *dhfr*, *dhps*

## Abstract

**Background:**

Non-synonymous mutations in *dhfr* and *dhps* genes in *Plasmodium vivax* are associated with sulfadoxine–pyrimethamine (SP) resistance. The present study aimed to assess the prevalence of point mutations in *P. vivax dhfr* (*pvdhfr)* and *P. vivax dhps* (*pvdhps*) genes in three countries: Lao PDR, India and Colombia.

**Methods:**

Samples from 203 microscopically diagnosed vivax malaria were collected from the three countries. Five codons at positions 13, 57, 58, 61, and 117 of *pvdhfr* and two codons at positions 383 and 553 of *pvdhps* were examined by polymerase chain reaction-restriction fragment length polymorphism methodology.

**Results:**

The largest number of 58R/117 N double mutations in *pvdhfr* was observed in Colombia (94.3 %), while the corresponding wild-type amino acids were found at high frequencies in Lao PDR during 2001–2004 (57.8 %). Size polymorphism analysis of the tandem repeats within *pvdhfr* revealed that 74.3 % of all the isolates carried the type B variant. Eighty-nine per cent of all the isolates examined carried wild-type *pvdhps* A383 and A553.

**Conclusions:**

Although SP is not generally used to treat *P. vivax* infections, mutations in *dhfr* and *dhps* that confer antifolate resistance in *P. vivax* are common. The data strongly suggest that, when used primarily to treat falciparum malaria, SP can exert a substantial selective pressure on *P. vivax* populations, and this can lead to point mutations in *dhfr* and *dhps*. Accurate data on the global geographic distribution of *dhfr* and *dhps* genotypes should help to inform anti-malarial drug-use policies.

## Background

The antifolate drug combination sulfadoxine–pyrimethamine (SP) has been used as an anti-malarial treatment because of its low cost and relative safety. The therapeutic targets of SP in malaria parasites are dihydropteroate synthase (DHPS) and dihydrofolate reductase (DHFR) enzymes. Molecular epidemiology studies have revealed that the point mutations in the malaria parasite’s *dhfr* and *dhps* genes, which confer resistance to SP, change the amino acid residues around the active sites of the enzymes they encode [1]. Progressive accumulation of point mutations is associated with increasing levels of drug resistance in malaria parasites. In *Plasmodium falciparum*, a mutation at codon 108 of DHFR, which confers low-grade pyrimethamine resistance, is observed first, followed by additional mutations that induce high-level pyrimethamine resistance at codons 51 and 59 [2]. Accumulation of *P. falciparum* DHFR (PfDHFR) mutations at codons 16, 51, 59, 108, and 164 is associated with progressively increasing levels of pyrimethamine resistance [3]. Patients infected with *P. falciparum* isolates carrying these point mutations are more likely to experience SP treatment failure [4, 5]. Point mutations have been identified in *Plasmodium vivax* DHFR (PvDHFR) at codons 51, 58 and 117, which correspond to PfDHFR positions 51, 59 and 108 [6–8]. Additionally, PvDHFR mutations at codons 57 and 61 in combination with those at codons 58 and 117 have been reported to correlate with SP treatment failure [9, 10].

Point mutations in PfDHPS codons 436, 437, 540, 581, and 613 have been found to be associated with sulfadoxine resistance [11], and in *P. vivax* homologous PvDHPS mutations have been described at codons 382, 383, 512, 553, and 585. Mutations in PvDHPS codon 383 and 553, which correspond to PfDHPS positions 437 and 581, have been found at high prevalence in Thailand [12, 13]. Parasites carrying multiple mutations in *pvdhfr* and *pvdhps* are cleared more slowly from the patient’s blood during SP treatment [12]. Because monitoring of anti-malarial drug susceptibility in *P. vivax* parasites in vitro is limited by the difficulties in culturing this parasite species, molecular markers for SP resistance are an important tool for evaluating SP resistance patterns in *P. vivax* populations. The well-described patterns of mutations related to drug resistance in *P. falciparum* and *P. vivax* have led to the development of the polymerase chain reaction-restriction fragment length polymorphism (PCR–RFLP) technique as a molecular surveillance tool for predicting SP drug resistance in specific geographical areas.

Molecular studies on point mutations in *pvdhfr* and *pvdhps* have been reported in many countries. Mutations in *pvdhfr* that confer SP resistance are found to be distributed wildly in Thailand [7, 13, 14], Myanmar [15], Cambodia [16], Vietnam [17], Indonesia [18, 19], Papua New Guinea [20], the Philippines [21], Bangladesh [21], Nepal [21], Pakistan [22], China [23], and India [8, 24, 25]. Although data on *pvdhfr* and *pvdhps* genotypes are available for many Southeast Asian countries, such data remain limited in some *P. vivax*-endemic areas, notably Lao PDR and South America. Reports from South America have shown that high prevalence of PvDHFR double mutations in codon 58 and 117 were found in Colombia [26, 27] while most of the samples from French Guiana have multiple point mutations in PvDHFR [28]. In this study, the prevalences of point mutations in *pvdhfr* and *pvdhps* were studied in three countries: Lao PDR, Colombia and India. India and Colombia are two countries in which *P. vivax* is present as the predominant malaria infection-causing species [29–31]. The prevalence of antifolate anti-malarial drug resistance in India is relatively high, with many recent reports of mutations in *pvdhfr* and *pvdhps*, whereas information on drug resistance mutations in Colombia remains limited. *Plasmodium vivax* infections are common in rural Laos [32], but no published information is available on *pvdhfr* and *pvdhps* mutations.

In this study, point mutations in *pvdhfr* and *pvdhps* were investigated as a tool for surveillance of SP resistance. The pattern of mutations present in each study area provide valuable molecular information on antifolate drug resistance, and this information may be useful for epidemiological mapping of drug-resistant vivax malaria.

## Methods

### Parasite isolates and DNA extraction

Blood samples were collected from symptomatic *P. vivax*-infected patients as either whole blood or dried blood spots obtained from three different countries: Lao PDR, India and Colombia. *Plasmodium vivax* isolates (n = 136) from Lao PDR were collected between 2001 and 2004, and 45 samples were collected between 2008 and 2012. In India, the 117 samples were collected from patients who attended the malaria clinic at the Calcutta School of Tropical Medicine, Kolkata between April 2003 and September 2004. Fifty-three *P. vivax* isolates from Colombia were collected between 2001 and 2004 from five areas (Amazonas, Buenaventuno, Red Tumaco, Red Equapi, and Quibdó). All of the clinical isolates from the three countries were reported as single-species infections of *P. vivax* as determined by light microscopic examination of Giemsa-stained blood smears. This study received ethical approval from the Faculty of Tropical Medicine, Mahidol University (MUTM 2011-055-01).

Genomic DNA was extracted from all the isolates using a commercially available DNA extraction kit (QIAGEN, Germany) following the manufacturer’s instructions. DNA was extracted from 200 μl of whole blood or one dried blood spot on Whatman 3MM filter paper (1 cm diameter) in a final elution volume of 100 μl. DNA samples were kept at −20 °C before use.

### PCR analysis of parasite species

Confirmation of the microscopic detection of *P. vivax* and detection of other cryptic species that might be in the samples were achieved using a nested PCR amplification assay based on the *SSU rRNA* gene [33].

### PCR–RFLP of PvDHFR and PvDHPS

Nested or semi-nested PCR amplification of PvDHFR was carried out using a method described previously [8]. Five point mutations (I13L, F57I/L, S58R, T61 M, S117 N/T) were examined by RFLP using the corresponding restriction enzymes for each specific position in the *pvdhfr* gene [8]. In the case of PvDHPS, two positions (A383G and A553G) were investigated by PCR–RFLP, following the protocol described previously [12]. All the DNA fragments obtained from the RFLP analysis were subjected to electrophoresis on 2 % agarose gels before visualization on an ultraviolet transilluminator after ethidium bromide staining.

### Detection of size polymorphisms in PvDHFR

The tandem repeat region between nucleotides 262 and 309 of the *pvdhfr* gene was PCR amplified using a protocol published previously [7]. Allelic size variants, which were assigned as described previously [7], were designated A, B and C from the largest to the smallest in base pairs, respectively (range, ca. 230–280 bp).

## Results

Of the 203 *P. vivax* malaria samples from the three countries, nested PCR detected nine samples containing mixed infections with *P. falciparum*. Mixed infections were present at high frequency in Lao PDR (2001–2004, eight out of 98 samples), while in India mixed infections accounted for one of 32 samples, and there were no mixed infections in the 28 Colombian samples. The result have shown demographic history of vivax population and their resistant background in each region, which can be part of further consideration for malaria eradication programme.

**Fig. 1:**
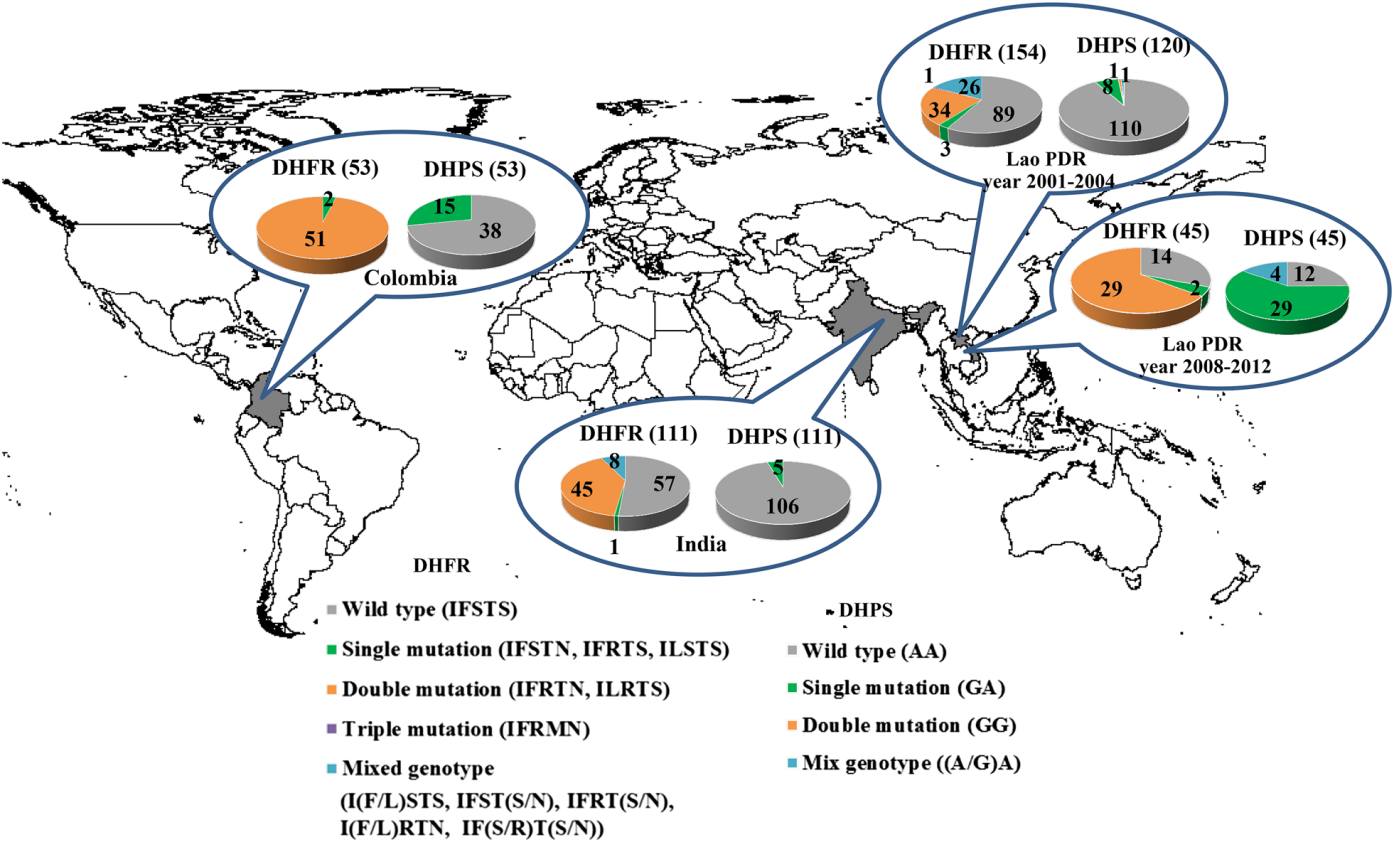
Distribution of PvDHFR and PvDHPS mutations in Colombia, India and Lao PDR. The numbers next to DHFR and DHPS represented the number of samples. The number count for each mutation type is shown on each graph. Amino acids in bracket after each haplotype of DHPS corresponds to PvDHPS codon 383 and 553. For DHFR, there are 5 codons corresponds to PvDHFR codon 13, 57, 58, 61 and 117

Genotype polymorphisms within PvDHFR were examined in five codons at positions 13, 57, 58, 61, and 117 (Fig. 1). Most of the samples collected from Lao PDR during 2001–2004 (89 out of 154) contained wild-type sequences at these five codons, compared with the 58R and 117 N double mutations found at high frequencies during 2008–2012 (29 out of 45 samples). High numbers of double mutations were also found within Colombia (51 out of 53) and India (45 out of 111). The number of haplotypes based on these five residues in PvDHFR was limited for Colombia, India and Lao PDR during 2008–2012 in comparison with the samples from Lao PDR during 2001–2004. The total numbers of PvDHFR haplotypes for Colombia, India and Lao PDR during 2008–2012 were three, five and three, respectively. The majority of the non-wild-type genotypes identified in all three regions had double mutations at codons 58R and 117 N. Double mutations in 58R/117 N were found at high frequency in the Colombian isolates (51 out of 53 samples) and in the Lao PDR isolates between 2008 and 2012 (29 out of 45 samples). It was noticed that mixed genotype infections of wild-type and mutant parasites were found at high frequency in the isolates from Lao PDR (2001–2004, 26 of 154), while there were no mixed genotype infections in Colombia.

**Fig. 2:**
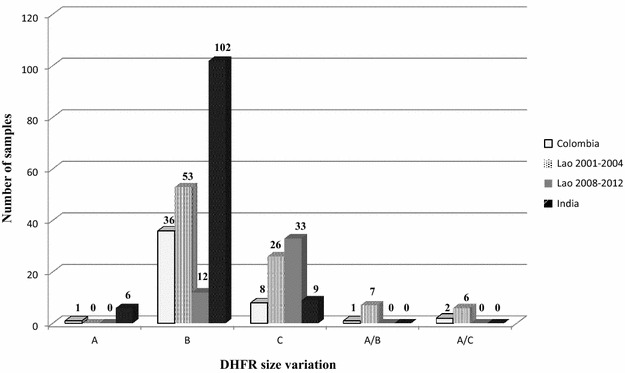
Distribution of DHFR allelic size variations in *Plasmodium vivax* parasites from Colombia, India and Lao PDR, based on the tandem repeats within *pvdhfr.* The numbers on *top* of each graph represent the number of samples. *Capital letters* on the x-axis represent the allelic types

Size polymorphisms in *pvdhfr* were investigated in 48, 92, 45, and 117 samples from Colombia, Lao PDR (2001–2004), Lao PDR (2008–2012) and India, respectively. The samples from all three countries contained a high prevalence of the parasite allelic variant type B, except Lao PDR (2008–2012), which had a high frequency of the allelic type C. No mixed allelic types were found in India and Lao PDR (2008–2012), but mixed A/B and A/C allelic types were found in seven and six samples, respectively, from Lao PDR (2001–2004), while one and two samples each of mixed allelic types, A/B and A/C, respectively, were identified in the Colombian samples (Fig. 2). Part of samples that carry allelic variant type A, B, and C with PCR product size approximating 280, 250, and 230 bp, and their sequences around the central repeat unit were subsequently determined. Deduced amino acid sequences of type A, B, and C are GGDNTS GGDNTH GGDNTH GGDNAD, GGDNTS GGDNTH GGDNAD, and GGDNTS GGDNAD, respectively.

Point mutations in PvDHPS were investigated at two positions: A383G and A553G. The majority of the samples from all three countries contained wild-type residues at these two positions (Fig. 1). The 383G single mutation was found in all three countries at frequencies of 28.3 % (Colombia), 6.7 % (Lao PDR, 2001–2004), 64.4 % (Lao PDR, 2008–2012) and 4.5 % (India). Since the mutation at 383G was often found with 382S in Colombia and associated with SP resistance [27], eleven samples carrying 383G and 9 wild type samples were submitted for sequencing. It was found that all 20 samples carry wild type amino acid at codon 382. Double mutations at 383G and 553G and mixed genotypes of the wild-type and the position 383 mutation were found in one sample for each type within the samples from Lao PDR (2001–2004).

**Fig. 3:**
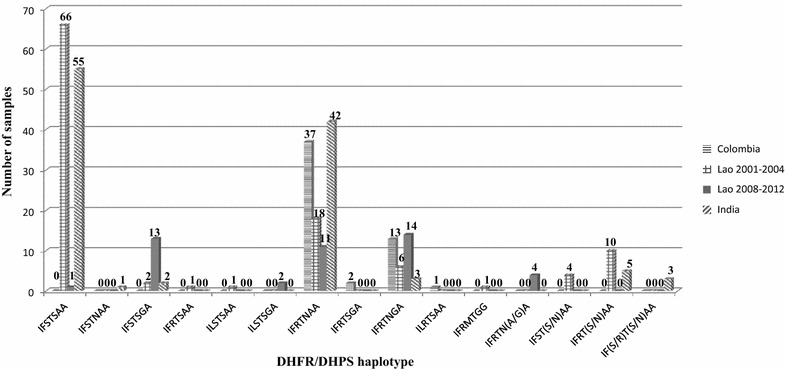
Distribution of DHFR-DHPS haplotypes found in Colombia, India and Lao PDR. The numbers on *top* of each graph represent the number of samples. The x-axis shows the various haplotypes. The amino acid sequence for each haplotype corresponds to PvDHFR codons 13, 57, 58, 61, 117, PvDHPS codons 383 and 553

Haplotype classification based on five point mutations in PvDHFR and two point mutations in PvDHPS was achievable for 53, 109, 45, and 111 samples from Colombia, Lao PDR (2001–2004), Lao PDR (2008–2012) and India, respectively. Figure 3 shows the number of haplotypes identified in samples from each country for the 15 haplotypes identified herein. Samples from Lao PDR (2001–2004) and India showed a high prevalence of the wild-type IFSTSAA haplotype at 60.6 and 49.5 %, respectively. A double point mutation (58R/117 N) in PvDHFR in combination with wild-type PvDHPS IFRTNAA was found with high frequency in Colombia at 69.8 %. Comparing the two time periods for Lao PDR revealed that the period 2008–2012 contained a high frequency of mutant alleles. It was found that the double 58R/117 N mutation in PvDHFR and the single 383G mutation in DHPS predominated in Lao PDR between 2008 and 2012, with a value of 31.1 %.

## Discussion

In this study, the distribution of mutant alleles in *pvdhfr* and *pvdhps* was examined in three geographical regions with different levels of *P. vivax* endemicity and differing histories of recent drug usage. India and Colombia have a relatively high prevalence of *P. vivax* infections (60–65 %) compared with *P. falciparum* infections [30, 31, 34], while Lao PDR has a relatively low prevalence of *P. vivax* infections (20–25 %) compared with *P. falciparum* infections [32]. Perhaps because of this, the prevalence of cryptic *P. falciparum* co-infections in patients presenting with *P. vivax* infections in Lao PDR has been reported to be 10 % [35], a figure consistent with the data from the current study.

Size polymorphism regarding the GGDN repeat unit in PvDHFR was investigated and the result showed high prevalence of allelic variant type B in most of the samples. The patterns of PvDHFR point mutations in each allelic type were considered and the result showed no significant association. This result was similar to the previous findings where PvDHFR point mutations did not show significant association to any allelic type [6, 16, 19]. As shown from the crystal structure, the GGDN repeat region was located outside the binding pocket of PvDHFR enzyme and thus thought to be nonessential for substrate binding [36].

Surveillance of SP resistance was investigated with PCR-RFLP to determine specific point mutation in five codons of PvDHFR and two codons of PvDHPS. This information provides some points of view for epidemiological mapping of SP resistance and their background situations in vivax malaria. *Plasmodium vivax* parasites from India were found to have a high frequency of *dhfr* and *dhps* mutations [37]. Recent studies of PvDHFR genotypes in India have reported high frequencies of 58R and 117 N double mutations [37], which is similar to the results for PfDHFR mutations [38, 39]. The results from this study showed that *P. vivax* parasites from India carried the wild-type DHFR allele at approximately the same high frequency (51.4 %) as they carried the 58R/117 N double mutation (40.5 %), suggesting that the double mutation and wild-type alleles are still common in India. SP is not the drug of choice for treatment of *P. vivax* infections in India because *P. vivax* is still susceptible to chloroquine [30]. Therefore, the appearance of the double mutation in PvDHFR is likely to be caused by the use of SP to treat *P. falciparum* infections, thereby exerting a drug selection pressure on the sympatric *P. vivax* population. The data suggest that use of antifolate combinations for *P. vivax* infections should be carefully validated. The therapeutic efficacy of the SP drug combination should be investigated.

Analysis of the *pvdhfr* mutations in Colombia revealed a high frequency of the 58R and 117 N double mutation, similar to previous observations [27]; this double mutation in DHFR was also found at high frequency in *P. falciparum* [40]. It has been proposed that use of SP in Colombia has exerted an evolutionary selection pressure on both *P. falciparum* and *P. vivax*. Therefore, the SP combination must be carefully validated for its efficacy.

No scientific data for *pvdhfr* mutations in Lao PDR have been published. Infections caused by *P. falciparum*, the predominant malaria parasite species in Lao PDR, were found to contain double and triple mutations in DHFR [41–43]. This survey in Lao PDR encapsulated two time periods: 2001–2004 and 2008–2012. From 2001–2004, most of the *P. vivax* population still carried the wild-type allele for *dhfr*. The number of mutations increased and double mutations were found in *pvdhfr*, while a single mutation was found in *pvdhps* during 2008–2012. As *P. vivax* is often found to co-exist with the more prevalent *P. falciparum* in Laos, it is likely that *P. vivax* has been under heavy SP pressure before the switch to artemisinin-based combination therapy (ACT). It has been speculated that the change in treatment policy to ACT was timely. However, artemisinin resistance has already emerged and spread in Southeast Asia. Epidemiological surveys aimed at identifying artemisinin-resistant parasites in Lao PDR are essential for assessing the impact of ACT policy in this country.

## Conclusion

These data suggest strongly that SP used to treat *P. falciparum* exerts a substantial selective pressure on the *P. vivax* population, leading to the selection of drug resistance-conferring mutations in *pvdhfr* and *pvdhps*.

## Abbreviations

ACT: artemisinin-based combination therapy
DHFR: dihydrofolate reductase
DHPS: dihydropteroate synthase
PCR–RFLP: polymerase chain reaction-restriction fragment length polymorphism
PvDHFR: *Plasmodium vivax* dihydrofolate reductase
PvDHPS: *Plasmodium vivax* dihydropteroate synthase
SP: sulfadoxine–pyrimethamine
